# Nutritional Analysis of Red-Purple and White-Fleshed Pitaya (*Hylocereus*) Species

**DOI:** 10.3390/molecules27030808

**Published:** 2022-01-26

**Authors:** Şule Hilal Attar, Muhammet Ali Gündeşli, Ipek Urün, Salih Kafkas, Nesibe Ebru Kafkas, Sezai Ercisli, Chunfeng Ge, Jiri Mlcek, Anna Adamkova

**Affiliations:** 1 Department of Horticulture, Faculty of Agriculture, University of Çukurova, Balcali, Adana 01330, Turkey; sulehilal35@gmail.com (Ş.H.A.); ipek-016@hotmail.com (I.U.); skafkas@cu.edu.tr (S.K.); ebruyasakafkas@gmail.com (N.E.K.); 2 Department of Plant and Animal Production, Nurdagı Vocational School, Gaziantep University, Gaziantep 27310, Turkey; maligun4646@gmail.com; 3 Department of Horticulture, Agricultural Faculty, Ataturk University, Erzurum 25240, Turkey; 4 Institute of Botany, Jiangsu Province and Chinese Academy of Sciences, Nanjing 210014, China; 18761866172@163.com; 5 Department of Food Analysis and Chemistry, Faculty of Technology, Tomas Bata University in Zlin, Vavreckova 275, CZ-76001 Zlin, Czech Republic; mlcek@utb.cz (J.M.); aadamkova@utb.cz (A.A.)

**Keywords:** pitaya (*Hylocereus* spp.) sugars, phenolic compounds, volatiles, HS-SPME/GC-MS

## Abstract

Pitaya is one of the most preferred and produced tropical fruit species recently introduced to the Mediterrranean region in Turkey. Due to its nutritional fruits with high economic value, the popularity of pitaya increases steadily in Turkey as an alternative crop. No detailed nutritional analysis has been undertaken in Turkey so far on fruits of the pitaya species. In this study, we determined and compared some nutritional parameters in fruit flesh of two pitaya (dragon fruit) species (*Hylocereus polyrhizus*: Siyam and *Hylocereus undatus*: Vietnam Jaina) grown in the Adana province located in the eastern Mediterranean region in Turkey. The individual sugars, antioxidant activity, total phenolic content, phenolic compounds and volatiles were determined for the first time in Turkey on two pitaya species. The results showed that total phenol content and antioxidant capacity are notably higher in red-fleshed fruits than white-fleshed ones and the predominant phenolic compound in fruits of both species was quercetin. The total sugar content and most of the phenolic compounds in fruits of two pitaya species were similar. A total of 51 volatile compounds were detected by using two Solid Phase Micro Extraction (SPME) fibers, coupled with Gas Chromatography Mass Spectrometry (GC-MS) techniques, and more volatile compounds were presented in the white-fleshed species. Total phenolic content (TPC) of the red-fleshed and white-fleshed pitaya species were 16.66 and 17.11 mg GAE/100 g FW (fresh weight). This study provides a first look at the biochemical comparison of red-fleshed and white-fleshed pitaya species introduced and cultivated in Turkey. The results also showed, for the first time, the biochemical content and the potential health benefit of *Hylocereus* grown in different agroecological conditions, providing important information for pitaya researchers and application perspective.

## 1. Introduction

Pitaya, known as dragon fruit, belongs to the genus *Hylocereus* of the *Cactaceae* family. The species is native to southern Mexico, the Pacific side of Guatemala and Costa Rica, and El Salvador. There are three species in the genus *Hylocereus*. These are *Hylocereus guatemalensis (Hg)*, *Hylocereus polyrhizus* (*Hp*) and *Hylocereus undatus (Hu)*. These species and their hybrids are grown commercially worldwide [1,2]. There are distinct morphological differences in stem, flower and fruit characters in the genus *Hylocereus* spp. [3]. The fruit is white, crimson, or dark red, or pale yellow, depending on the variety, and the flesh is interspersed with small black seeds [4]. *Hp* is the common red-fleshed species and known to be a super fruit with high antioxidant capacity due to its dark red flesh. The fruit is highly decorative, with a bright red skin adorned with green scales. *Hu* is a vine-like cactus commonly grown as a nocturnal ornamental and fruit crop. The fruit is highly decorative, with a bright red skin adorned with green scales. The flesh is white, juicy and delicious [3,5]. Pitaya fruits are consumed fresh or processed into juice, jellies, marmalades, jams, wine and beverages [6,7,8]. 

Fifteen years ago, pitaya was practically unknown but today is popular in the European market and in the global market, including Vietnam, Colombia, Mexico, Costa Rica, the USA (Florida and California) and Nicaragua [9]. Pitaya is considered a new and promising fruit. Australia, India, Thailand, Taiwan, Malaysia, the Philippines, Indonesia, Israel, and Turkey have begun to grow it [10,11,12] and it has been commercialized and consumed in many parts of the world [13,14,15]. 

Pitaya has great potential as a new crop for Mediterranean growers: it consumes little water, and it adapts well to the high temperatures present in the greenhouse. Moreover, the market shows increasing demand for new, healthy exotic fruits, and the fruit of these crops are worldwide appreciated as a super fruit.

Fruit quality is a term that denotes a degree of excellence, a high standard or value, for both the producer and the consumer. These distinctive quality features are contributed to by color, appearance, taste-aroma (flavor) and nutritional values. There are a few studies on this subject in pitaya fruit. Volatile compounds and primary metabolites are important indicators of fruit quality and flavor that play an important role in determining product acceptability by consumers. Consumers determine their preferences according to these features [12,16,17]. Increasing awareness of the benefits of plant products to ensure adequate intake of macronutrients, dietary fiber, vitamins, micronutrients and phytochemicals that are very important to human health is increasingly widespread among the population. It has been reported that pitaya, which is very rich in bioactive substances such as betacyanins, phenolic compounds, polysaccharides and terpenoids, can be consumed as a preventive agent against cancer, obesity, type 2 diabetes and other metabolic disorders [18,19,20]. Recently, the identification and content of these phytochemicals in different plant products are of great importance due to their various positive effects on human health. Last decade, with the increasing death rate resulting from cancer, cardiovascular and heart diseases, more epidemiological studies have suggested that the healthy food and fruits may decrease the risk of these diseases. Since pitaya does not contain cholesterol, saturated fat or trans-fat, it helps in regulating blood pressure and keeping cholesterol under control when consumed regularly. In addition, due to the high fiber content, it is easier to remove toxic substances from the body and the blood sugar level is kept in balance [21,22,23,24]. In recent years, there has been great interest in using natural antioxidants, including fruits, in preventive medicine and in the food industry [12,17].

Due to their nutraceutical and beneficial properties, phytonutrients found in medicinal plants such as polyphenols (especially phenolic acids) are worth considering. These compounds are important bioactive secondary metabolites that have long been studied for their enhancement and prevention capacities and are therefore considered potential sources for pharmaceutical and medical applications. Pitaya has a moisture content of around 80%, protein content between 0.4 and 2.2 g, carbohydrate content between 8.5 g and 13.0 g, and its total sugar content is around 6.0 g per 100 g fresh weight [25,26]. Especially the red and white fleshed pitaya fruits are a rich source of phenolic compounds, vitamins (B1, B2, B3, C, niacin, pyridoxine and cobalamin), minerals (calcium, potassium, phosphorus, sodium, iron and zinc), proteins, fats, carbohydrates, sugars, fiber and volatiles [16,27,28,29]. It is also associated with high antioxidant capacity of bioactive compounds, such as polyphenols, flavonoids and betacyanins, that have important functions for human health [12,28,30,31,32]. Some epidemiological studies have shown that pitaya has various bioactivities such as antioxidant, anticancer, antimicrobial, anti-hyperlipidemic, anti-diabetic, hepatoprotective, and wound healing [6,12,17,19,33,34]. Therefore, pitaya appears to have many important compounds. Thus, the fruit not only has economic value but also has a high potential for application in the food and pharmaceutical industry. Additionally, the fruit peel, pulp and seeds of pitaya are used in the manufacture of different foods such as ice cream, yogurt, fruit juice, jelly and marmalade [2]. 

Carbohydrates are one of the most important macronutrients found in abundance in the different tissues of pitaya. In recent years, a number of studies have been carried out investigating the properties and levels of carbohydrates obtained in the different tissues of pitaya [35,36]. Sugars, together with phenolic components, play an important role in the sensory and nutritional quality of the product and may vary according to the cultivar, maturity stages, storage conditions, crop year and fruit development conditions. It has been reported that dried pitaya peels are a rich source of pectin and dietary fiber (60–80%) [37]. Red pitaya fruit is very rich in iron content. Scientists have proven that consuming red pitaya fruit juice drastically increases hemoglobin and erythrocyte levels in pregnant women. Thus, consuming it during pregnancy is an alternative treatment for anemia [38]. 

Jerônimo et al. [26] analyzed the flesh of the *H. undatus* and found that the predominant fatty acids were oleic, linoleic, and palmitic acid as 50.8%, 21.5% and 12.6% of the total fatty acid content, respectively. Similarly, Ariffin et al. [39] analyzed the oil extracted from red and white pitaya seeds and found a high content of essential fatty acids, namely linoleic (~50%) and linolenic (~1%) acid, and other fatty acids such as cis-vaccenic acid (~3.0%), palmitic acid (17.5%), and oleic acid (22.7%). The human health benefits of poly-and monounsaturated fatty acids have been documented and they help to reduce cholesterol and lipoprotein fractions [40,41]. It has also been reported that linoleic and alpha-linolenic acids are required to maintain cell membranes, brain function, and transmission of nerve impulses [26,42]. In recent years, pitaya fruits have greatly increased in popularity worldwide due to their attractive colors, sweetness, juicy and pleasant taste. This fruit has been recognized as the most beautiful of the *Cactaceae* family [43]. There are many species of the pitaya; however, the *Hylocereus undatus* is the best known and widely cultivated.

Due to having a red-purple color, pitaya is highlighted by global breeders for its rich polyphenol compounds and antioxidant capacities. Studies on pitaya are increasing in foreign countries but mostly focuses on processing technology such as wine, pigment extraction and optimization of fermentation conditions with some biochemical composition analysis. Volatiles are often key components that play a role in determining fruit flavor but have not been adequately studied in pitaya, and studies of pitaya fruit aroma components are comparatively scarce. However, the aroma components and sugars in pitaya fruit, which are particularly important for fruit wine, are relatively less studied. Nowadays, especially *Hu* or white-fleshed pitaya fruits are attracting more attention than other fruits because of their sensory properties, economic importance, and because they are high in antioxidants that help reduce many diseases [44,45].

As it is known, tropical fruits are difficult to grow outside of tropical regions. However, these fruits can be grown specially in microclimate conditions. In Turkey, some regions such as Mersin, Adana and Antalya have microclimatic conditions and are suitable for pitaya cultivation [2]. Therefore, pitaya is a very economical product for many traditional producers because its cultivation requires little or no investment. It can thus be considered as an alternative product with high commercial potential. However, many of the detailed fruit qualities and differences among different flesh colored pitaya remain unclear. As far as we know, there is limited information on the detection of volatile profiles of pitaya flesh. Similar to sugars, aroma is also one of the most important fruit quality parameters for consumer acceptance and gives objective information for further breeding studies.

In this study, we determined and compared some nutritional parameters in the fruit flesh of two pitaya species (*H. polyrhizus*: Siyam and *H. undatus*; Vietnam Jaina) which are grown in Turkey. The results provide some fruit quality information for the selection of suitable pitaya species for growers in Turkey, especially the volatile compounds that will also set up a reliable database for the pitaya industry. 

## 2. Materials and Methods

### 2.1. Materials

Fruit materials were harvested from two pitaya species *Hp* and *Hu* from local producers in Adana/Turkey (20 August 2019). Both species have red outer peel and *Hu* and *Hp* had white and red flesh color, respectively. Fifteen randomly selected fruits for each species were harvested and transferred to the Instrumental Analysis laboratories of Cukurova University, Adana, Turkey under cold chain conditions. 

### 2.2. Samples and Extracts Preparation

The fruits were peeled manually, and samples were divided into 3 groups for replicates; every 5 fruits were accepted as one replicate. Their flesh was homogenized using fruit extractor and immediately stored at −80 °C until analysis. These triplicated homogenized materials were used for further biochemical analysis. The same homogenate is used for sugars, phenolic compounds, volatile compounds, total phenol and antioxidant capacity analysis. Pitaya flesh were homogenized using a kitchen blender. 

### 2.3. Total Antioxidant Capacity (TAC)

The free radical scavenging activity (RSA) of pitaya flesh was measured according to the DPPH (2,2-diphenyl-1-picrylhydrazyl) assay by Brand-Williams et al. [46] with slight modifications. A methanolic extract (methanol/water; 70:30; *v*/*v*) was used for both total antioxidant capacity and total phenol analysis. Briefly, 0.06 μM of ethanolic DPPH was freshly prepared. Then, 1950 μL of DPPH was added to 50 μL of pitaya fruit sample. The mixture was shaken for 1 min and kept in the dark for 30 min at room temperature. The radical scavenging activity was measured at 515 nm for 5 min intervals using a Multiscan GO microplate spectrophotometer. The solvent was used as blank and the % radical scavenging activity of the pitaya flesh extract was calculated using the following formula:

DPPH radical scavenging activity (%) = [(Control absorbances − Sample absorbances) / Control absorbances] × 100

### 2.4. Total Phenol Contents (TPC)

Total phenol content was spectrophotometrically determined using the Folin–Ciocalteu procedure described by Spanos and Wrolstad [47] with a slight modification. The analysis was completed with a UV/VIS spectrophotometer (Thermo Fisher Scientific, Vantaa, Finland). Briefly, 9 mL of 70% methanol were added to 1 mL of the fruit sample. The mixture was centrifuged at 5500 rpm for 10 min. A volume of 50 μL of supernatant was added to 250 μL of Folin–Ciocalteu reagent. Afterward, 750 μL 20% (*w*/*v*) Na_2_CO_3_ were supplemented, and it was incubated 2 h at room temperature. Then, the absorbance was measured at 760 nm against a blank. The same procedure was applied to gallic acid standards (0–1 mg/mL intervals). The total amount of phenolic substances was calculated by using the calibration curve y = 0.839x + 0.0622 (r^2^ = 0.998), prepared with the gallic acid standards and results are expressed as mg GAE/100 g fresh weight (FW) of pitaya flesh.

### 2.5. Sugar Analysis

Glucose, fructose, xylose and total sugar contents in homogenized pitaya samples were determined using the HPLC technique according to the method developed by Cristosto [48]. Glucose, fructose and xylose (Sigma-Aldrich, St. Louis, MO, USA) are used as external standards (15–2500 ppm) for sugar analysis. Before analysis, frozen fruit samples were thawed at 25 °C. One g of homogenized fruit sample was added to 4 mL of ultrapure water (Millipore Corp., Bedford, MA, USA). The reaction mixture was placed in an ultrasonic bath and sonicated at for 15 min and then centrifuged at 5500 rpm for 15 min and it was filtered before HPLC analysis (Whatman nylon syringe filters, 0.45 µm, 13 mm, diameter). Triplicate analysis was completed and HPLC (Shimadzu, Prominence LC-20A) RID (Refractive Index) detector and Coregel-87C (7.8 × 300 mm) HPLC column were used. Separations were performed at 70 °C at a flow rate of 0.6 mL/min. Elution was isocratic ultrapure water. The individual sugars were calculated according to their standards and expressed as percent fresh weight (FW). Calibration curves of all references were created and content was determined according to for the calibration curves for glucose y = 266.56x + 0; fructose y = 253.36x + 0; xylose y = 256.48x + 0 (Those r^2^ = 0.999)

### 2.6. Phenolic Compounds

For the extraction and hydrolysis procedure of phenolics, 0.5 g of homogenized samples were used boiled using a reflex unit for 1 h [49]. After it was cooled, the mixture was filtered and made up to 10 mL with the extraction solvent (acetone:water; 3:1). These samples were directly used for HPLC analyses. The liquid chromatographic apparatus (HPLC, Shimadzu LC-20A, Tokyo, Japan) consisted of an in-line degasser, pump, and controller coupled to a photodiode array detector equipped with an automatic injector (20 µL injection volume) interfaced to a PC running ChemStation chromatography manager software. Separations were performed on a 150 mm × 4.6 mm i.d., 5 µm, reverse-phase Nucleosil C18 analytical column (Supelco, Bellefonte, PA, USA) operating at room temperature with a flow rate of 1 mL /min. Detection was carried out with a sensitivity of 0.1 a.u.f.s. between the wavelengths of 280 and 360 nm. Elution was affected using a nonlinear gradient of the solvent mixture 2.5% HCOOH in water (solvent A) and 2.5% HCOOH in acetonitrile (solvent B). The composition of B was increased from 5 to 13% in 15 min, increased to 15% in 5 min and to 30% in a further 5 min and held for 3 min, increased to 45% in 4 min and held for 3 min, increased to 90% in 5 min and held for 5 min, and then returned to initial conditions in 5 min.

Gallic acid, myricetin, caffeic acid, *p*-coumaric acid, ellagic acid, quercetin and kaempferol (Sigma-Aldrich), were used as phenolic standards. Gallic acid, caffeic acid, *p*-coumaric acid, ellagic acid, myricetin, quercetin and kaempferol were dissolved in methanol at a concentration of 1000 ppm samples were directly injected into the reverse phase chromatography column. Five dilute solutions from these stock solutions were used for calibration curves and calibration curves were obtained for caffeic acid y = 49,033.3x + 0, for ellagic acid y = 31,923x + 0, for quercetin y = 21,275x + 0 and for kaempferol y = 27,358.73x + 0, respectively. Recoveries were measured by extracting the recovered amounts of pure substances added to frozen pitaya samples before the experiment. Three replicates from each sample were used for HPLC analyses. All the samples and standards were injected three times.

### 2.7. Volatile Compounds 

In this study, one-gram homogenate pitaya was weighed and 1 mL of CaCl_2_ added during 30 min at 40 °C incubation time. The SPME fiber 85 μm PDMS/DVB (Polydimethylsiloxane/Divinylbenzene; blue) and SPME fiber 100 μm PDMS (Polydimethylsiloxane; red) were compared. The adsorbed flavor compounds of the pitaya fruits were analyzed using a Shimadzu GC-2010 Plus Gas chromatography mass spectrometer (GC-MS). HP-Innowax Agilent column (30 m × 0.25 mm i.d., 0.25 µm thickness) was used and helium was the carrier gas. The GC oven temperature was kept at 40 °C and programmed to 260 °C at a rate of 5 °C/min, and then kept constant at 260 °C for 40 min. The injector temperature was at 250 °C. The MS was taken at 70 eV. The mass range was *m*/*z* 30–400. A library search was carried out using the commercial Wiley, Nist and Flavor GC-MS Libraries. The mass spectra were also compared with those of reference compounds and confirmed with the aid of retention indices from published sources. Relative percentage amounts of the separated compounds were calculated from total ion chromatograms on the computer. We use alkanes C7 to C24. By looking at the retention time of the aroma compound, the RI value of that aroma compound is calculated according to the formula below, using the retention times of the alkane compounds that are between the Retntion times or exit times of the two alkane compounds.

RT: Retentıon time: exit time of aroma compounds
Ca: Exit time of alkane containing “a” carbons
Ca + 1: Exit time of alkane containing “a + 1” carbon

RI = 100 × [ (RT − Ca)/(Ca + 1 − Ca) + a]

### 2.8. Statistical Analysis

The statistics of biochemical analysis were done using Mann Whitney U test and Principal Component Analysis (PCA). Mann Whitney U test explores the differences between two independent groups and is a non-parametric test which means it is useful when the dependent variable is not normally distributed. Our research attempted to see whether the biochemical compounds in the flesh of red and white pitaya differed significantly by employing the Mann Whitney U test. PCA is a method that explains the variance-covariance structure of a set of variables. As a product of PCA, biplot graphs are useful to show interunit distances, as well as variances and correlations of variables [50]. Data inspections were done using biplots. In addition, correlation analysis was also used to see the interrelations of the observations. Mann Whitney U test was performed by using SPSS, and PCA and correlation analysis were performed through XLSTAT software.

## 3. Results

Among the fruit quality characteristics, phenolic compounds, sugars, total phenol, antioxidant capacity and volatile compounds are the most important parameters [22,26,51,52,53,54,55,56,57,58,59,60,61,62,63,64]. 

### 3.1. Total Phenolic Content and Antioxidant Capacity

Total phenolic content (TPC) and antioxidant capacity (TAC) of red-purple and white-fleshed pitaya species were shown in Table 1. TPC of red and white-fleshed pitaya were 16.66 to 17.11 mg GAE/100 g FW, respectively (Table 1). The red-purple fleshed pitaya fruits have a higher value of TPC than the white-fleshed ones (Table 1). Abirami et al. [3] reported that TPC of the different pitaya varieties were between 32.5 and 42.5 mg GAE/100 g FW. Previously pitaya fruit was reported to have significantly higher total phenolic content compared to the other tropical fruits [51]. Choo and Yong [10] reported that TPC in the pitaya fruit flesh is 28.65 mg GAE/100 g FW. Esquivel et al. [16] revealed that TPC varied from 9.12 to 13.3 mg/g in different Hylocereus spp. fruits. Nurliyana et al. [5] also reported that Hu and Hp varieties had TPC between 3.75 and 19.72 mg GAE/100 g. Pasko [52], reported that the TPC in all samples was higher in water than methanol extracts and red-fleshed pitaya had the highest TPC, followed by white and yellow fleshed pitaya. The results for TPC in the fruits were consistent with Perez-Loredo [53], and Garcia-Cruz [54], who observed higher concentration of these compounds in red-fleshed than in white ones. Mello et al. [55] found that the TPC was s 40.68 mg GAE/100 g FW in pitaya peel. Antioxidant assays including DPPH and ABTS were used to measure the radical scavenging ability, while FRAP and TAC assays were used to determine the reducing power of samples. The DPPH assay is one of the non-specific free radical scavenging assays as it measures free radicals scavenged by both phenolic and non-phenolic compounds, including gallic acid [56]. The findings indicated that the red-fleshed pitaya fruits have the higher TAC (46.81%) while white-fleshed has the lower TAC (38.16%) as we compare two samples (Table 1). Abirami et al. [3reported that the TAC in fruits of the different pitaya varieties were found to be 36 and 70.1% in the flesh. Wu et al. [57] studied the TAC of red pitaya. According to them, peel and flesh were both rich in phenolics and good sources of antioxidants. Some of the authors previously reported that the antioxidant capacity of pitaya flesh is due to the presence of both betalains and phenolic compounds, which have the ability to donate their electrons and to scavenge the ABTS cation radical, as Trolox does [58,59]. However, the interactions that occur between different types of compounds within each group, to express a particular antioxidant capacity, are not fully understood. The TAC of the most abundant betalain present in the fruit does not always correlate with the TAC expected for the whole fruit, but rather is the result of the interaction of all the antioxidant compounds it contains. Both betalains and phenolic compounds could influence the TAC of pitaya fruit. Esquivel et al. [16] reported that antioxidant capacity of purple-fleshed pitaya was based mostly on betalains and other non-betalainic phenolics such as gallic acid and acetylcoumarins. García-Cruz et al. [17] also reported that the TAC in pitaya pulp was highly variable and found between 2.41 and 9.21 μmol Trolox/g FW determined by the ABTS. Halimoon and Abdul Hasan [27] also reported that *H. undatus* exhibited the highest RSA activity (63.44%) of DPPH. Chaves et al. [60] and Beltran-Orozco et al. [61] reported high antioxidant capacity in their studies in red, white and yellow-fleshed pitaya fruits, respectively. The antioxidant capacity of pitaya fruit has been noted to be higher than some other tropical fruits such as mango, lychee, longan and papaya [62]. Oxygen radical absorbance capacity (ORAC) varies in the range of 8.80–11.30 µmol/g depending on the variety and bioactive component profile [58]. The findings of our study on red-purple and white-fleshed pitaya are generally compatible with the findings of other researchers, but some values were found to be higher and some lower. This variation in the results can be attributed to geographic and seasonal variations. It is also dependent on the genotypes in between different Hylocereus species, cultivation conditions, treatments and applied analysis methodologies.

### 3.2. Sugar Content

In the present study, the contents of carbohydrates (sucrose, glucose, xylose, fructose, total sugar) in fruits of two pitaya species were measured. Data related to individual sugars and total sugars are shown in Table 1. There were significant differences in terms of sugar contents of experimental species in the study (Table 1). Among many phytochemical compounds found in pitaya fruits, the sugar composition affects the perceived fruit sweetness [34,51,59,63]. As seen from these results, the most predominant sugars in these two species were glucose and followed by fructose, and then xylose (Table 1). Concerning the sugar contents of the red-fleshed specie, glucose (7.52%) was found to be higher than the contents of fructose (3.70%) and xylose (0.03%). On the other hand, in the white-fleshed specie, glucose (5.22%) content was lower, but fructose was higher than in the red-fleshed specie. Glucose was found to be the dominant sugar in both species and high glucose value was detected in the red-fleshed specie. In addition, the total sugar contents of the red-fleshed specie (11.25%) had higher than white-fleshed one (10.24%). Jamilah et al. [64] previously reported that glucose content of red-fleshed pitaya was 4.15% and fructose content was 0.86%. In another study, Jeronimo et al. [26] stated that total sugar was detected as 5.92% in pitaya fruit flesh.

### 3.3. Phenolic Compounds

Phenolic compounds are an excellent source of antioxidants that play an important role in protecting human health. These compounds also contribute to the taste, color, and health benefits of plants [65,66,67]. Phenolic compounds play a major role in contributing to overall antioxidant capacity [68]. There are very limited and almost no reports of the presence of individual phenol compounds in fruits of cacti species. Here, identification and quantification of individual phenolic compounds of *Hu* (red pitaya with white flesh) and the *Hp* (red pitaya with red flesh) performed by HPLC and the contents of phenolic compounds are provided in Table 2. According to the results, seven phenolic compounds were identified. Quercetin was dominant—3.43 mg/100 g in red-fleshed pitaya and 3.09 mg/100 g in white-fleshed pitaya. Gallic acid, kaempferol, ellagic acid, *p*-coumaric acid, caffeic acid and myricetin content were 0.17 mg/100 g; 0.21 mg/100 g, 0.15 mg/100 g, 0.16 mg/100 g, 0.12 mg/100 g and 0.33 mg/100 g in red-fleshed pitaya fruits, respectively. In white-fleshed pitaya fruits, gallic acid, kaempferol, ellagic acid, *p*-coumaric acid, caffeic acid and myricetin content were 0.16 mg/100 g; 0.26 mg/100 g, 0.10 mg/100 g, 0.16 mg/100 g, 0.13 mg/100 g and 0.47 mg/100 g, respectively (Table 2).

Jamilah et al. [64] reported 150.46 mg of phenolic compounds (betalains, gallic acid, and betacyanins) per 100 g dry weight in the *Hylocereus* species. Esquivel et al. [16] reported that gallic acid was observed in all the genotypes, with higher amounts in ‘Rosa’ and lowest in ‘Orejona’ pitaya varieties.

The same authors implied that gallic acid was identified for the first time in pitaya fruits and the phenolic profiles generally differed between genotypes. Several flavonoids such as catechin, epicatechin, quercetin, myricetin, kaempferol, and rutin were detected and quantified in pitaya seeds by Adnan et al. [69]. According to their results, catechin (3.60 mg/g dw), quercetin (1.31 mg/g dw) and myricetin (0.63 mg/g dw) were found to be the major flavonoids, respectively. Tenore et al. [70] reported that red pitaya fruits contain gallic acid, pyrocatheunic, vanillic acid, caffeic acid, and *p*-coumaric acid. In another study, Lim et al. [11] reported that the *p*-coumaric acid, syringic acid, caffeic acid, vanillic acid, pyrocathehunic and gallic acid in flesh and peels of red pitaya fruits were 0.78 mg/100 g, 0.08 mg/100 g, 0.08 mg/100 g, 0.64 mg/100 g, 0.93 mg/100 g and 0.25 mg/100 g, respectively. Suleria et al. [56] reported higher content of quercetin, myricetin, kaempferol in pitaya than our results. Pitaya flesh (pulp) was also found to be abundant in phenolic acids while quercetin was dominantly detected. Previously, flavonoids including kaempferol and quercetin derivatives were detected and quantified in pitaya fruit peels as well [71,72].

### 3.4. The Difference of Volatile Compounds between Red- and White-Fleshed Pitaya

The aroma in fruits is an important parameter to attract consumers and commercial market competition in pitaya fruit [67]. In addition to fruit flavor, volatile compounds have also been reported in some studies to play important roles in pitaya fruit aging, biotic-abiotic stress factors, and responses to plant disease [29,51,67,73,74]. In the present study, 39 volatile compounds were detected in pitaya fruit flesh by HS-SPME/GC-MS techniques (Table 3). Regarding their chemical composition in the samples tested, they were shown to be complex mixtures of many components. Table 3 shows the compounds identified, retention time and percentage obtained by GC-MS. Two different assayed SPME fibers (PDMS/DVB, 85 μm, blue) and (PDMS, 100 μm, red) were used for the detection of volatile compounds.

A total of 39 volatile compounds and six chemical groups including the alcohols, terpenes, ketones, aldehydes, esters, acids and other compounds were identified (Table 3). Similarly, Wu et al. [74] and Wu et al. [75] identified a total of 84 and 49 volatile compounds in pitaya fruit by using DVB/CAR/PDMS fiber (Divinylbenzene/Carboxen/Polydimethylsiloxane; Gray). These compounds also mainly include aldehydes, esters, alkanes, ketones, terpenes and alcohols.

The detailed compound names and content values are presented in Table 4. In the present study, we compared the extraction capabilities of both SPME fibers in red and white-fleshed pitaya. According to Table 4, the PDMS fiber extracted a total of 32 compounds and that of PDMS/DVB was 32. The PDMS fiber is preferred for the extraction of non-polar analytes, whereas the polar polyacrylate (PA) fiber is more suitable for the extraction of polar analytes.

Mixed coating fibers, containing divinylbenzene (DVB) copolymers, templated resin (TPR) or carboxen (CAR) increase retention capacity. Thus, PDMS/DVB and CAR/DVB fibers can be more efficacious for the extraction of low molecular weight volatile compounds [76,77]. Although both PDMS/DVB and PDMS fibers could extract a similar number of volatile compounds, the detailed compounds differ greatly, 12 volatile compounds were extracted by both PDMS and PDMS/DVB fibers; these compounds are described in Table 4.

These results also showed that the PDMS and PDMS/DVB could independently extract 15 and 14 volatile compounds, respectively (Table 4). Obenland et al. [73] detected nineteen aroma volatiles such as aldehydes, alcohols, ketones, hydrocarbons using Solid Phase Micro Extraction (SPME) in six *Hylocereus* cultivars (Cebra, Rosa, Lisa, San Ignacio, Mexicana, and Physical Graffiti) grown in California. They also found that aldehydes accounted for more than 90% of the total volatile amount. In another study, Quijano-Célis et al. [78] identified 121 volatiles such as alcohols, terpenes, paraffins, acids, esters, and ketones from *H. megalanthus* using the aroma extract dilution analysis method, and various other compounds using solvent extraction followed by concentration. They also performed aroma extract dilution analysis (AEDA) to identify nine fragrance active compounds that could potentially affect pitaya flavor.

There are some similarities and variations between previous studies and our study, and these differences can be explained by differences in the examined pitaya tissues and the variability of the extraction methods applied, plant material type and maturity stage, growing regions and climatic conditions. The previous studies conducted with horticultural plants indicated that biochemical and volatile content greatly varied among cultivars, geographical conditions, growing conditions, etc. [79,80,81,82,83,84,85,86,87,88,89,90,91,92].

As a result, both SPME fibers could extract most of the volatile compounds such as alcohols, ketones, aldehydes, acids, easter and several other compounds. Here, we also found the different preferences of PDMS and PDMS/DVB fibers in the extraction of volatile compounds in the pitaya fruit; only the PDMS fiber could specially extract the terpenes (l-Limonene) in both red- and white- fleshed compared to that of PDMS/DVB. The technique of SPME has been widely used to detect the composition of volatile compounds in foods and drinks because this method is organic solvent-free, time-saving and low cost compared to traditional extraction methods. The method could seperate and quantify cis-and trans-isomers. PDMS (100µm polydimethylsiloxane) for volatiles and PDMS/DVB (65 µm polydimethylsiloxane/divinylbenzene). The SPME method suitably recovered most compounds reported to impart characteristic flavors/aromas in pitaya fruits. SPME offers experimental flexibility and the ability to discover more compounds and address flavor quality differences among pitaya species (Table 3 and Table 4).

The Mann–Whitney U test was used to test the difference between groups. This nonparametric test was chosen due to the small number of observations and the lack of normal data distribution. According to the results, a significant difference was observed in ellagic acid, myricetin and kaempferol between the white and red-fleshed species at a 10% significance level. In detail, ellagic acid was significantly higher in red, and myricetin and kaempferol were higher in White-fleshed pitaya samples. There is also a difference at the 10% significance level in antioxidant capacity, which was seen more in white-fleshed pitaya (Table 5).

### 3.5. Principal Component Analysis (PCA)

PCA was used to determine the correlation between the various parameters. In a biplot, which is a product of PCA, the length of the lines approximates the variances of the variables. The longer line indicates higher variance. The cosine of the angle between the lines approximates the correlation between the variables they represent. The closer the angle is to 90 or 270 degrees, the smaller the correlation. An angle of 0 or 180 degrees reflects a correlation of 1 or −1, respectively. Very distant breakpoints in the variable line direction indicate high values, while breakpoints on the variable line extended along the beginning represent low values. Finally, the distance between two points approximates the Euclidean distance between two observations in multivariable space. Observations that far apart have a high Euclidean distance and vice versa [50].

**Figure 1:**
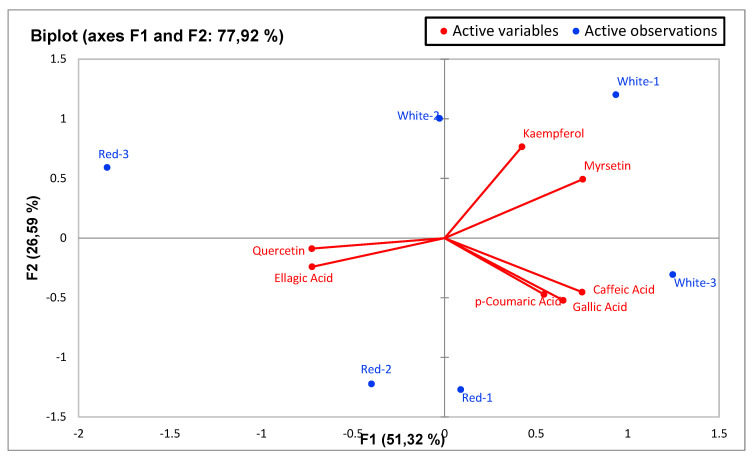
Biplot graph (scores and loading plots) obtained from Principal Component Analysis for phenolic compounds in red-white pitaya.

**Figure 2:**
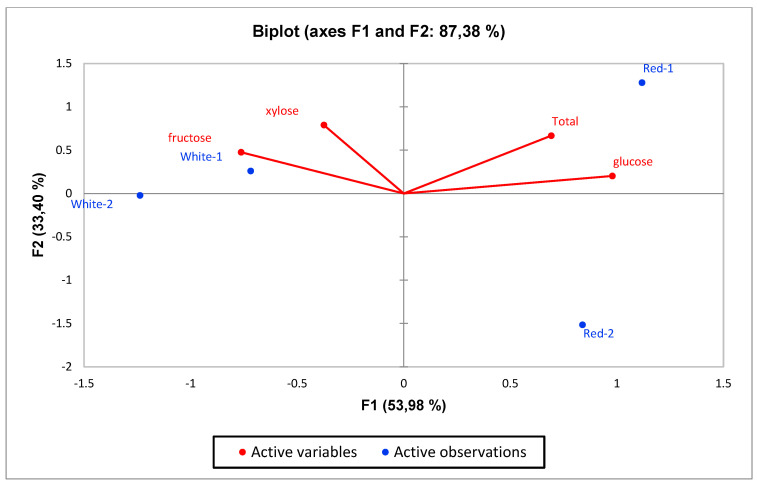
Biplot graph (scores and loading plots) obtained from Principal Component Analysis for sugars in red-white pitaya.

**Figure 3:**
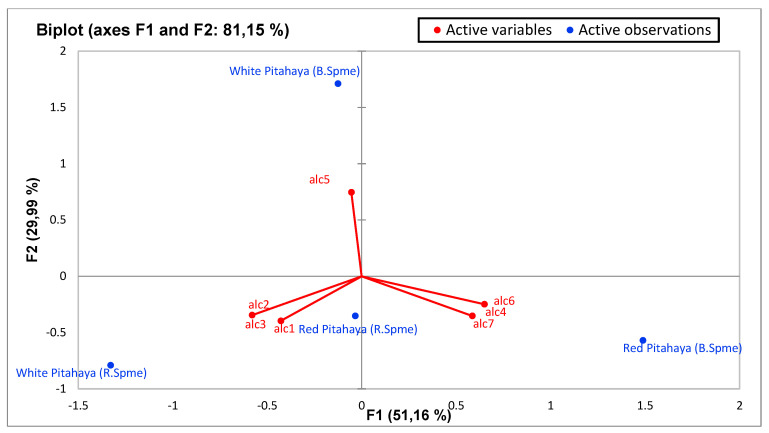
Biplot graph (scores and loading plots) obtained from Principal Component Analysis for Alcohols from volatile compounds in red-white pitaya. alc1: 1-Hexadecanol, alc2: 1-Tetradecanol, alc3: dimethyl-Silanediol, alc4: <2-ethyl->Hexanol, alc5: Lauryl alcohol, alc6: Pentadecanol, alc7: Tridecyl alcohol.

**Figure 4:**
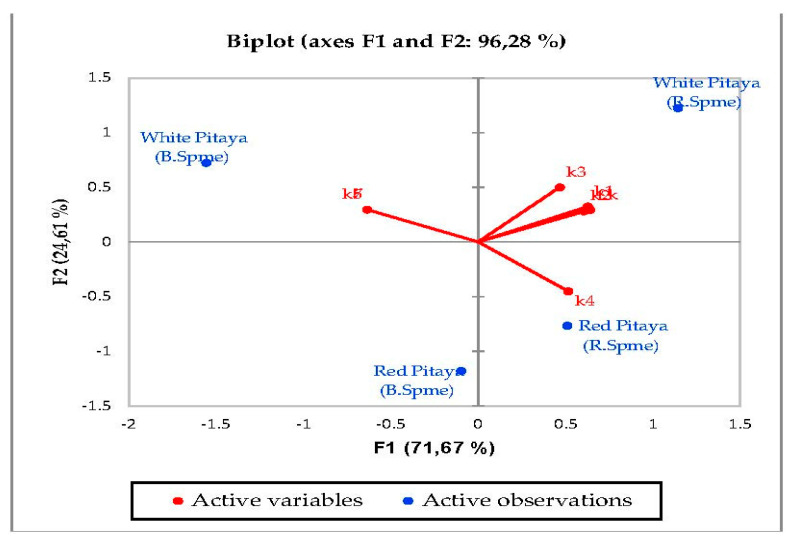
Biplot graph (scores and loading plots) obtained from Principal Component Analysis for ketones from volatile compounds in red-white pitaya. k1: 1-hydroxy- 2-Propanone,k2: 1-(2-furanyl)- Ethanone, k3: 2,3-Butanedione, k4: 2-Propanone, k5: 5-hexyldihydro- 2(3H)-Furanone, k6: diphenyl- Methanone, k7: <delta->Dodecalactone; R.Spme: Red Spme; B.Spme: Blue Spme.

**Figure 5:**
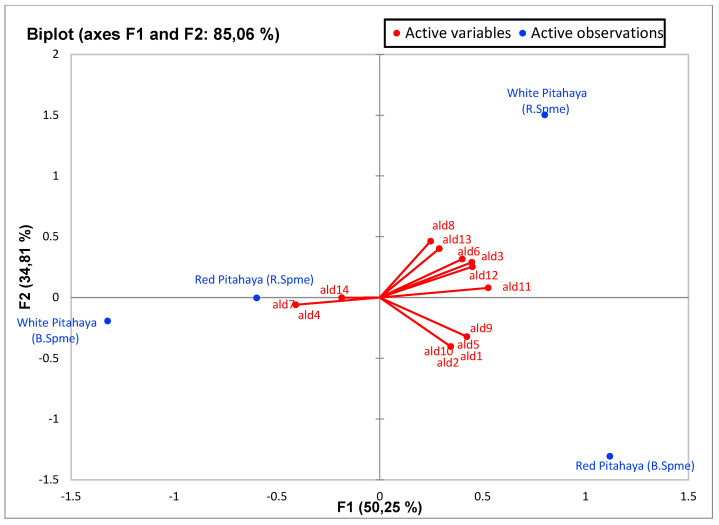
Biplot graph (scores and loading plots) obtained from Principal Component Analysis for aldehydes from volatile compounds in red-white pitaya. ald1: 2 octenal, ald2: 2,4 nonadıenal, ald3: 2-Decenal, (E)-, ald4: 2-furancarboxaldehyde, ald5: 2-heptenal, (Z)-, ald6: 2-oxo- Propanal, ald7: 3-methyl-Benzaldehyde, ald8: caprylaldehyde, ald9: hexanal, ald10: Non-2(E)-enal, ald11: nonanal, ald12: octanal, ald13: Trans-2-Dodecenal, ald14: trans-2-Undecenal.

**Figure 6:**
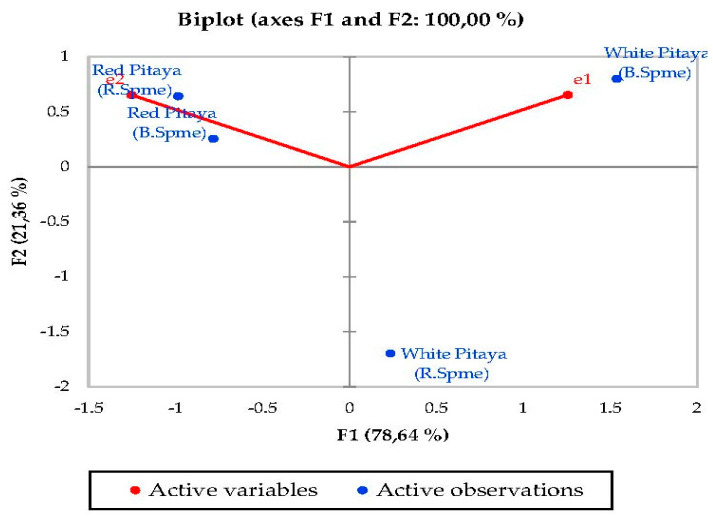
Biplot graph (scores and loading plots) obtained from Principal Component Analysis for esters from volatile compounds in red-white pitaya. e1: 1,2-Benzenedicarboxylic acid, diethyl ester, e2: Acetic acid, ethyl ester.

**Figure 7:**
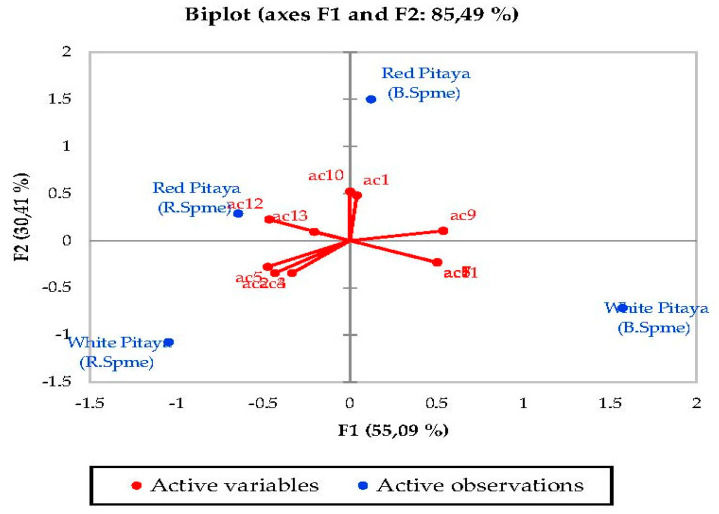
Biplot graph (scores and loading plots) obtained from Principal Component Analysis for acids from volatile compounds in red-white pitaya. ac1: 2-Acetonyl-3-cyano-2,3-dimethylcyclobutane-1-carboxylic acid, ac2: acetic acid, ac3: anhydride acetic acid, ac4: caprylic acid, ac5: Carbamic acid, monoammonium salt, ac6: decanoic acid, ac7: dodecanoic acid, ac8: heptanoic acid, ac9: hexanoic acid, ac10: nonanoic acid, ac11: octanoic acid, ac12: pentadecanoic acid, ac13: tetradecanoic acid.

**Figure 8:**
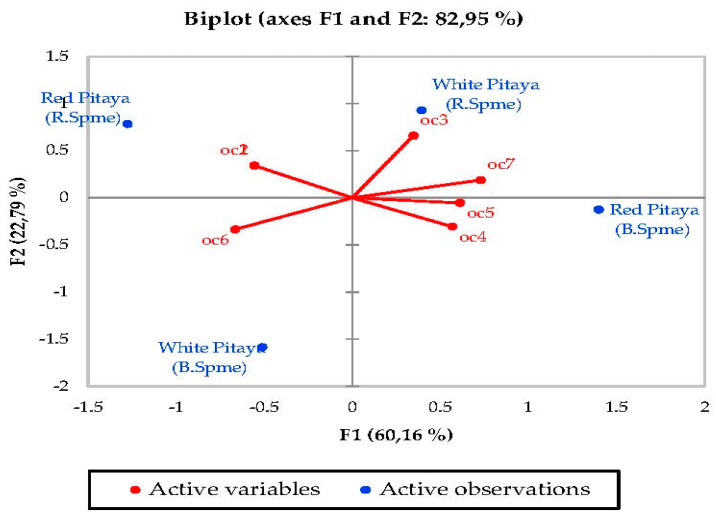
Biplot graph (scores and loading plots) obtained from Principal Component Analysis for other compounds from volatile compounds in red-white pitaya. oc1: methyl isocyanide, oc2: 1-methyl->Piperidine, oc3: 2,6-bis(1,1-dimethylethyl)-4-methyl- Phenol, oc4: 1,1′-oxybis- Octane, oc5: 2,6,10,14-tetramethyl- Hexadecane, oc6: tetranitro- Methane, oc7: Tridecane.

Principal component analysis (PCA) was performed to statistically reveal the effect of individual phenol compounds and sugar contents on red and white-fleshed pitaya (Figure 1, Figure 2, Figure 3, Figure 4, Figure 5, Figure 6, Figure 7 and Figure 8). In other words, as the outputs of the PCA analyses, biplot graphs enabled us to visualize the interrelations between variables and observations.

According to the biplot graph of the phenolics obtained as a result of Principal Component Analyses (PCA), PC1 (the first principal component) explained 51.32% of the total variance, while PC2 explained 26.59%. The variance that the two factors can explain is 77.92% in total; which phenolics were higher in observations and the relationships between phenolics were given visually in the Figure 1. In detail, the highest quercetin and ellagaic acid levels were observed in red-fleshed samples (especially in red 2 and red 3), while highest levels of kaempferol and myrsetin were detected in white samples. Besides, white-3 sample contained the highest gallic acid, caffeic acid and *p*-coumaric acid levels. According to the PCA analysis related to sugars, PC1 explained 53.98% of the total variance, while PC2 (the second principal component) explained 33.40%. The total explained variance is 87.38% by the first two principal components. According to these findings, while xylose and fructose are closely related, total sugar and glucose are also closely related to each other. On the other hand, these two sugar groups were inversely correlated with the others, while fructose and xylose were higher in whites, total sugar and glucose were higher in reds (Figure 2). The highest levels of fructose and xylose were found in white-fleshed samples, while the highest levels of total sugar and glucose were observed in red-fleshed samples. In other words, red and white-fleshed samples created two groups in terms of sugar content. The relationships between variables and observations in volatile aroma substances in white and red-fleshed pitaya fruit are given in the figures with virtual and biplot graphs obtained as a result of PCA (Figure 3, Figure 4, Figure 5, Figure 6, Figure 7 and Figure 8). For the alcohols, PC1 can explain 51.16% of the total variance while PC2 can explain 29.99%. 81.15% of the total variance can be explained by the first two factors (Figure 3). White pitaya (B. Spme) had the highest alcohols—5 I—while red-fleshed (R. Spme) and white-fleshed (B. Spme) also had higher alcohols at 1-2-3 values. In addition, alcohols 4-6-7 were the highest in red-fleshed fruits (R. Spme). For the Ketones, PC1 explained 71.67% of the total variance and PC2 explained 24.61% in peduncle samples. These two components may explain 96.28% of the total variance (Figure 4). Red-fleshed (R. Spme) had higher k 5-6-7 while white-fleshed (B. Spme) had higher k 1-2-3 and t.k.

For the aldehydes, PC1 explained 50.25% of the total variance, PC2 explained 34.81% in peduncle samples. These two components can explain 85.06% of the total variance (Figure 5). Ald7-4-14 was found to be the highest in red-fleshed (R. Spme), and white-fleshed (B. Spme) had a similar trend. Aldehydes 1, 2, 5, 9 and 10 were the highest in red-fleshed (R. Spme) whereas the rest of the aldehyde were the highest in white-fleshed (B. Spme).

For esters, PC1 explained 78.64% of the total variance and PC2 explained 21.36% in peduncle samples. These two components may explain 100% of the total variance (Figure 6). E2 was the highest in red-fleshed pitaya while e1 was the highest in white-fleshed (B. Spme). For acids, PC1 explained 55.09% of the total variance and PC2 explained 30.41% in peduncle samples. These two components may explain 85.50% of the total variance (Figure 7). Acids 12-13 were the highest in the red-fleshed pitaya (R.Spme).

Other compounds, PC1 explained 60.16% of the total variance, PC2 explained 22.79% in samples. These two components may explain 82.95% of the total variance (Figure 8). As a result, the variability of PCA results in all volatile compounds was found to be quite high, with the total variation ranging between 21.36% and 100.00%. All these figures and tables are a summary of the variables studied in this experiment. The result of the PCA analysis confirmed some important differences between total antioxidant and phenol and individual phenol compounds and sugars in different tissues of pitaya. In general, the variability of PCA results in all biochemical compounds was found to be quite high, with the total variation ranging between 26.59% and 100%. At the same time, all tissues analyzed generally had higher phenolic compound content in pitaya flesh. Finally, the red and white-fleshed pitaya are considered to be interesting raw material with important phenol compounds, and these compounds are particularly effective in human health.

## 4. Conclusions

Today, *Hylocereus* *undatus*, or pitaya fruit with red skin, attracts attention all over the world due to its white and red flesh, sensory properties and economic importance. The results of this study showed that pitaya flesh (pulp) has many aromatic compounds. The fruits are also rich in phenolic compounds and offer high antioxidant capacity. Our results have shown that pitaya fruit is a good source of bioactive phytochemicals that can provide good health effects. Furthermore, volatiles can be identified depending on the fiber used during the extraction. The SPME method is a convenient and accurate sampling technology for qualitative and quantitative analysis of volatile compounds in pitaya fruits. This situation increases the importance of the consumption of pitaya fruit for human health. No such study has been conducted on pitaya species studied in this region to date. This study, which can shed light on future studies, is also important and has a distinct value in this respect.

## Figures and Tables

**Table 1:** The content of sugars, total phenol, total antioxidant capacity of pitaya species (*Hylocereus* spp.).

Variety	Sugar				Total Phenol (mg/GAE 100 g)	DPPH%
	Glucose	Xylose	Fructose	Total		
Red-fleshed	7.52 ± 0.74	0.03 ± 0.04	3.70 ± 0.50	11.25 ± 1.27	17.11 ± 0.38	46.81 ± 2.93
White-fleshed	5.22 ± 0.39	0.05 ± 0.02	4.97 ± 0.48	10.24 ± 0.85	16.66 ± 0.56	38.36 ± 0.2.67

**Table 2:** Phenolic contents of two pitaya species (*Hylocereus* spp.) (mg/100 g).

Phenolic Contents	Red-Fleshed	White-Fleshed
Gallic Acid	0.17 ± 0.02	0.16 ± 0.03
Caffeic Acid	0.13 ± 0.02	0.12 ± 0.02
*p*-Coumaric Acid	0.16 ± 0.07	0.16 ± 0.04
Ellagic Acid	0.10 ± 0.03	0.15 ± 0.02
Myricetin	0.33 ± 0.02	0.47 ± 0.03
Quercetin	3.43 ± 0.16	3.09 ± 0.10
Kaempferol	0.21 ± 0.04	0.26 ± 0.03

**Table 3:** Volatile profiles of red and White-fleshed pulp pitaya species using red and blue SPME fibers by HS-SPME/GC/MS techniques. Retention Time (R.T) percent (%).

RI	R.T.	Compound Name	Red SPME (PDMS)		Blue SPME (PDMS/DVB)	
			Red Pitaya Pulp	White Pitaya Pulp	Red Pitaya Pulp	White Pitaya Pulp
		Alcohols				
1777	40.861	1-Hexadecanol	6.54	4.17	-	-
1587	36.229	1-Tetradecamol	-	6.69	-	-
1027	19.111	2-ethyl-hexanol	-	-	2.6	-
1472	33.177	Lauryl alcohol	-	-	-	37.17
1777	40.866	Pentadecanol	-	-	2.09	-
1586	36.188	Tridecyl alcohol	8.96	-	4.68	-
		Total Alcohols	15.5	10.86	9.37	37.17
		Terpenes				
989	17.713	l-Limonene	2.23	2.70	-	-
		Total Terpenes	2.23	2.70	0	0
		Ketones				
850	12.483	1-(2-furanyl)-Ethanone,	3.24	4.11	-	-
1456	32.730	5-hexyldihydro- 2(3H)-Furanone,	-	-	-	1.63
1781	40.952	diphenyl-methanone,	-	-	-	1.08
1714	39.369	delta-dodecalactone	-	-	-	1.30
		Total Ketones	3.24	4.11	0	4.01
		Aldehydes				
996	17.990	2 Octenal	-	-	6.84	-
1144	23.200	2,4 Nonadienal	-	-	1.56	-
1192	24.783	(E)- 2-Decenal	2.2	10.13	2.76	-
771	9.563	2-Furancarboxaldehyde	17.37	-	-	-
898	14.288	(Z)-2-Heptenal	-	-	5.11	-
1100	21.724	3-methyl benzaldehyde	-	-	-	1.37
1011	18.540	Caprylaldehyde	-	2.08	-	-
746	8.669	Hexanal	19.36	17.23	32.85	-
1158	23.658	2(E)Nonenal	-	-	2.75	-
1044	19.721	Nonanal	2.23	8.13	4.05	-
947	16.131	Octanal	-	6.34	1.8	-
1289	27.865	Trans-2-dodecenal	-	7.69	0.98	0.57
1173	24.159	trans-2-Undecenal	-	-	-	0.86
		Total Aldehydes	41.16	51.60	58.70	2.80
		Esters				
1643	37.628	1,2-Benzenedicarboxylic acid, diethyl ester	-	-	-	29.9
		Total Esters	0	0	0	29.9
		Acids				
1194	24.851	Caprylic acid	-	2.58	-	-
1551	35.285	Decanoic acid	-	-	-	3.36
1792	41.201	Dodecanoic acid	-	-	-	1.8
1321	28.818	Heptanoic acid	-	-	-	0.81
922	15.222	Hexanoic acid	8.07	5.84	6.74	6.58
1194	24.852	Nonanoic acid	2.75	-	6.65	-
1102	21.787	Octanoic acid	4.37	4.7	2.35	13.05
1845	42.401	Pentadecanoic acid	12.14	6.46	3.53	-
1658	37.993	Tetradecanoic acid	2.69	-	-	-
		Total Acids	30.02	19.58	19.27	25.6
		Other compounds				
767	9.430	<1-methyl->Piperidine	2.63	-	-	-
1438	32.206	2,6-bis(1,1-dimethylethyl)-4-methyl- Phenol	5.2	8.49	3.05	-
1582	36.100	1,1′-oxybis- octane,	-	-	2.05	0.53
1767	40.630	2,6,10,14-tetramethyl-hexadecane,	-	-	5.2	-
1248	26.573	Tridecane	-	2.64	2.37	-
		Total Other Compounds	7.83	11.13	12.67	0.53

-: not detected.

**Table 4:** Volatile profiles of between red and white pulp pitaya varieties detected by PDMS and PDMS/DVB fibers.

Category	PDMS- Red	PDMS- White	PDMS/DVB Red	PDMS/DVBWhite	PDMS	PDMS/DVB	Volatile Compounds Extracted by Both SPME Fibers
Alcohol	2	2	3	1	5	4	Tridecyl alcohol
Terpenes	1	1	0	0	1	0	-
Ketones	1	1	0	3	1	4	-
Aldehydes	4	6	9	3	13	9	(E)-2-Decenal, Hexanal, Nonanal, Octanal, trans 2 dodecenal
Esters	0	0	0	1	0	1	-
Acids	5	4	4	5	9	9	Hexanoic acid, Nonanoic acid, Octanoic acid, Pentadecanoic acid
Other compounds	2	2	4	1	3	5	2,6-bis(1,1-dimethylethyl)-4-methyl- Phenol, Tridecane
Total	15	16	20	14	32	32	13

Note: PDMS-Red/PDMS/DVB-Blue; volatiles extracted by PDMS and PDMS/DVB fiber in red and white pitaya.

**Table 5:** Mann–Whitney U test between groups.

	Gallic Acid	Caffeic Acid	*p*-Coumaric Acid	Ellagic Acid	Myricetin	Quercetin	Kaempferol	DPPH	Total Phenol
Mann–Whitney U	3.500	3.500	3.500	0.500	0.000	2.000	0.000	0.000	2.000
Wilcoxon W	9500	9.500	9.500	6.500	6.000	8.000	6.000	6.000	8.000
Z	−0.443	−0.443	−0.443	−1.798	−1.964	−1.091	−1.964	−0.964	−1.091
Asymp. Sig. (2-tailed)	0.658	0.658	0.658	0.072	0.050	0.275	0.050	0.050	0.275

## Data Availability

All-new research data were presented in this contribution.

## Sample Availability

Samples of the compounds are available from the authors.
